# Mega cisterna magna: a rare finding in a case of chronic occipital pain

**DOI:** 10.11604/pamj.2025.50.8.46083

**Published:** 2025-01-06

**Authors:** Anshu Tikariha, Pallavi Lalchand Harjpal

**Affiliations:** 1 Department of Neuro Physiotherapy, Ravi Nair Physiotherapy College, Datta Meghe Institute of Higher Education and Research, Wardha, Maharashtra, India

**Keywords:** Mega cisterna magna, cerebellomedullary cistern, posterior fossa malformation

## Image in medicine

Mega cisterna magna (MCM) is a rare posterior fossa cystic malformation. It is usually asymptomatic and often an incidental finding. We present a case of a 28-year-old male patient who was reported in the Neurophysiotherapy outpatient department with complaints of numbness and pain in the occipital region for 1 month. He had a history of mild episodes of the same symptoms before, in the past 4-5 years. Pain was scored according to the Numerical Pain Rating Scale (NPRS) as 7/10. On observation, there was evident flattening or loss of cervical lordosis. On examination, there was a restriction in flexion and side flexion on both sides at the end range, but it was pain-free. Mild tightness in the trapezius was present bilaterally. The flexion-rotation test and spurling were negative. On investigation, a magnetic resonance imaging (MRI) of the brain (A, B) suggested mild atrophic changes and a mega cisterna magna of A.P. diameter 19 mm with intact cerebellar vermis and no hydrocephalus. Magnetic resonance imaging of the spine suggested early degenerative changes, straightening of the spine along with diffuse disc bulge from C3-C6 disc level with mild narrowing of the spinal canal. He was prescribed cervical stabilisation exercises, stretching of the trapezius and TENS for pain management. After 1 week of treatment, the NPRS score was 4/10, with persistent numbness. While the findings suggest that cervical pathology could explain the patient’s occipital pain, further reporting, investigation, and correlation are needed to rule out the possibility of symptoms arising from mega cisterna magna.

**Figure 1:**
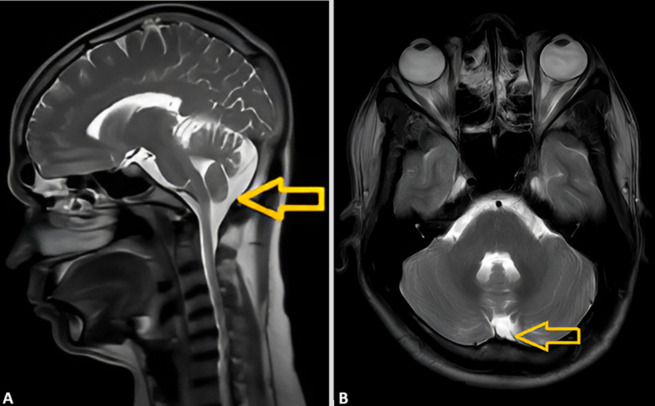
magnetic resonance image scans of the brain (T2-weighted) with; A) the sagittal, and B) axial planes show mega cisterna magna in the retro-cerebellar region as a fluid-filled space, marked with yellow arrows

